# Molecular mechanisms behind the temporal dynamics of LPS-induced inflammatory response in splenic phagocytes of the wall lizard *Hemidactylus flaviviridis*

**DOI:** 10.1242/jeb.251033

**Published:** 2026-05-28

**Authors:** Ananya Banerjee, Mamta Tripathy, Umesh Rai

**Affiliations:** ^1^ Department of Zoology, University of Delhi, New Delhi, Delhi 110007, India; ^2^ University of Jammu, Jammu Tawi, Jammu and Kashmir, 180006, India

**Keywords:** Reptiles, Wall lizard, Innate immunity, Lipopolysaccharide, Toll-like receptors

## Abstract

Our understanding of the molecular mechanisms underlying the reptilian immune response is strikingly limited despite their significant evolutionary placement between ectothermic anamniotes and endothermic amniotes. Hence, the current study attempted to understand the time kinetics of inflammatory pathways activated in immunostimulated splenic phagocytes of the wall lizard *Hemidactylus flaviviridis*. Our *in vitro* study revealed increased phagocytosis in splenic phagocytes at all time points (2, 8 and 12 h) of lipopolysaccharide (LPS) treatment. Further, qPCR analysis revealed LPS-induced modulation of immune-relevant genes in lizard splenic phagocytes. A biphasic increase in *tlr4* mRNA was observed at 2 h and 12 h of LPS treatment along with increased *tlr2* at all durations. Furthermore, in the early hours of LPS treatment, the expression of a signalling molecule (*myd88*) and transcription factors (*nfκb* and *ap1*) was upregulated. In addition, splenic phagocytes cultured with LPS for 2 h demonstrated increased expression of the pro-inflammatory cytokine *il1β* and antioxidant enzyme *catalase* genes with an inhibition of the anti-inflammatory cytokine *tgfβ* gene. While the heightened *il1β* and *catalase* expression reached basal levels 8 h post-LPS treatment, *tgfβ* expression returned to basal levels only after 12 h. Further, the current study also examined the expression of *arginase*, a marker of cell repair, which was significantly higher than respective controls at all time durations of LPS treatment. However, its maximal expression at 12 h is suggestive of increased cell repair activity during extended infection. Our results indicate activation of a pro-inflammatory and anti-oxidant response during the early hours of LPS exposure in splenic phagocytes, which dampens during later hours of bacterial challenge, raising the possibility of a shift to an anti-inflammatory and reparative response.

## Teaser

**Summary:** Investigation into the molecular mechanisms underlying the lipopolysaccharide-induced innate immune response in the reptile *Hemidactylus flaviviridis* reveals an initial pro-inflammatory and anti-oxidant response, followed by anti-inflammatory and reparative processes.

## INTRODUCTION

The vertebrate immune system, typically divided into innate and adaptive immunity, has evolved a diverse set of complex responses to combat pathogens (reviewed by Dempsey et al., 2003). Among chordates, while the earliest evidence of innate immunity can be found in the cephalochordates, a robust adaptive immune system first arose in jawed vertebrates, becoming increasingly specialised from fishes to mammals (reviewed by Schluter et al., 1999; reviewed by Buchmann, 2014). There is a notable shift in the predominance from innate to adaptive immunity during evolution from fishes to mammals. Among vertebrates, fishes (reviewed by Magnadóttir, 2006), amphibians (reviewed by Humphries et al., 2022) and reptiles (reviewed by Zimmerman, 2020) are more reliant on their innate immune system, while birds and mammals possess a more developed adaptive immunity (reviewed by Dempsey et al., 2003; reviewed by Mehrzad et al., 2024).

Among the ectothermic vertebrates, reptiles are the first true land dwellers. Their transition from an aquatic to a terrestrial mode of life must have required extensive immunological adaptations (reviewed by Zimmerman et al., 2010). While the reptilian spleen is made up of both red and white pulp, it lacks germinal centres (Kroese and van Rooijen, 1982; Leceta and Zapata, 1991), which necessitates their dependence on innate immunity. This gains further support from the fact that the reptilian humoral response is extremely slow and the antibody titres post-antigen exposure reach peak levels only after 6–8 weeks (reviewed by Zimmerman et al., 2010). The reptilian innate immune system is characterised by the presence of lysozymes, antimicrobial peptides, complement cascades as well as a wide repertoire of cells that provide non-specific immunity (reviewed by Zimmerman, 2020; Field et al., 2022), which enable reptiles to fight a wide array of pathogens such as protozoans, helminths, fungi, bacteria as well as parasitic arthropods present in their environments (reviewed by Mendoza-Roldan et al., 2020). Exposure to bacteria such as gram-positive *Mycoplasma* spp. or gram-negative *Aeromonas* spp. and *Salmonella* spp. is reported to cause pneumonia, rhinitis, as well as salmonellosis in reptiles (reviewed by Chinnadurai and Devoe, 2009; Pees et al., 2023). These infections lead to the development of common clinical signs of disease such as lethargy, anorexia, dysecdysis, colitis and osteomyelitis (reviewed by Chinnadurai and Devoe, 2009). However, the molecular mechanisms behind these responses remain underexplored, thus presenting a fascinating avenue of investigation. Within the class Reptilia, squamates represent the largest order with approximately 12,000 species (http://www.reptile-database.org). The high species richness of squamates and their ability to occupy diverse habitats exposes them to various pathogens in the environment, making them excellent models to study reptilian immunity. Therefore, the present study explored lipopolysaccharide (LPS)-mediated activation of innate immunity in a squamate reptile, *Hemidactylus flaviviridis*.

LPS is a glycolipid found on the outer cell membrane of gram-negative bacteria that confers pathogenicity and makes bacteria resistant to antimicrobial compounds (reviewed by Bertani and Ruiz, 2018). It is a potent and specific stimulator of the innate immune system that binds to the TLR receptor complex present on monocytes and macrophages, leading to cytokine production and complement system activation (reviewed by Farhana and Khan, 2021). LPS is extensively employed to study the molecular basis of immune responses activated upon bacterial infection across vertebrates including reptiles. It is known to activate innate immunity in squamates, as evident from the LPS-induced febrile response in lizards (Kluger et al., 1975; Bernheim and Kluger, 1976; Bernheim et al., 1978; Merchant et al., 2008; reviewed by Rakus et al., 2017) and snakes (Burns et al., 1996; reviewed by Rakus et al., 2017). Furthermore, LPS administration causes a heightened oxidative burst response in blood cells of painted dragon lizards (Tobler et al., 2015), while LPS treatment leads to an increased ratio of heterophils to lymphocytes in pygmy rattlesnake (Lind et al., 2020). Interestingly, previous investigations in our laboratory have demonstrated that wall lizard splenic phagocytes upon treatment with LPS secrete pro-inflammatory factors into the culture media (Mondal and Rai, 2001). The presence of pro-inflammatory factors in the spent culture media of the above experiment was validated by a thymocyte proliferation assay using rat thymocytes (Mondal and Rai, 2001), thus confirming that LPS is able to induce a pro-inflammatory response in the splenic phagocytes of *H. flaviviridis*.

Hence, the current study aimed to understand the molecular basis and time kinetics of the LPS-mediated inflammatory response in splenic phagocytes of the wall lizard *H. flaviviridis*. Phagocytes in mammals are the first responders of the innate immune system that identify pathogen-associated molecular patterns (PAMPs) using pathogen recognition receptors (PRRs), thereby initiating a cascade of neutralising reactions (reviewed by Marshall et al., 2018). Among the PRRs, the toll-like receptor (TLR) family member TLR4 actively recognises bacterial PAMPs such as LPS, leading to the activation of signal transduction pathways that eventually induce the production of inflammatory mediators such as cytokines (interleukins IL1β and IL6, interferon IFN-I, tumour necrosis factors TNFα, and others), chemokines and complement proteins as well as enzymes of oxidative stress (reviewed by Kawasaki and Kawai, 2014). Upon inflammation, these cells rapidly internalise the foreign particles by phagocytosis and perform lysosomal degradation of the antigens (reviewed by Uribe-Querol and Rosales, 2020). The current study hypothesised that in wall lizards, as in other vertebrates, LPS might stimulate splenic phagocytes of wall lizards and activate similar downstream signalling molecules to induce the expression of pro-inflammatory cytokines and markers of oxidative stress. To achieve this, the study employed *in vitro* experiments to explore LPS-mediated modulation in the expression of these immune-relevant factors in splenic phagocytes of the wall lizard *H. flaviviridis*.

## MATERIALS AND METHODS

### Animal maintenance

Adult female wall lizards, *Hemidactylus flaviviridis* Rüppell 1835, weighing 8–10 g were procured from a local supplier and housed in wooden cages (∼152×38×38 mm) lined with wire mesh on the top and sides to allow proper ventilation. The animals were acclimated to laboratory conditions of 12 h light:12 h dark at ambient temperature for a week before experimentation. During acclimation, lizards were provided with food (live houseflies) and water *ad libitum*. Before experimentation, the lizards were anaesthetised using carbon dioxide inhalation and killed by decapitation. The detailed protocol for animal experimentation and treatment was approved by the Institutional Animal Ethics Committee (IAEC) and guidelines of the Committee for the Purpose of Control and Supervision of Experiment on Animals (CPCSEA), Government of India, were followed.

### Reagents and culture medium

Fetal bovine serum (FBS) and Roswell Park Memorial Institute-1640 (RPMI-1640) media were procured from HiMedia Laboratories Pvt Ltd (Mumbai, India). The culture media was supplemented with 100 μg ml^−1^ streptomycin, 100 IU ml^−1^ penicillin, 40 μg ml^−1^ gentamicin (Ranbaxy India Ltd, New Delhi, India) and sodium bicarbonate [Sisco Research Laboratories (SRL) Pvt Ltd, Mumbai, India]. LPS from *Escherichia coli* O55:B5 (catalogue number L4524-5MG), diethyl pyrocarbonate (DEPC) and 3-(4,5-dimethyl-2-thiazolyl)-2,5-diphenyl-2*H*-tetrazolium bromide (MTT) were procured from Sigma Life Sciences (St Louis, MO, USA). Reagents used in RNA isolation, cDNA preparation and qPCR were TRIzol^TM^ reagent (Invitrogen™, Carlsbad, CA, USA), Wizard SV Gel and PCR Clean-Up System (Promega, Madison, WI, USA), Nuclease-Free Water (Invitrogen™), Verso cDNA synthesis kit (Thermo Fisher Scientific, Waltham, MA, USA) and PowerUp^TM^ SYBR^TM^ Green Universal Master mix (Applied Biosystems, Fisher Scientific). Giemsa stain and Baker's yeast (*Saccharomyces cerevisiae*) were procured from Thomas Baker (Chemicals) Pvt Ltd (TBCPL; Mumbai, India) and Solar Uni Ingredients (Delhi, India), respectively. Other routine chemicals such as hydrochloric acid (HCl), isopropanol, Triton X-100, glycerol and dibutylphthalate polystyrene xylene (DPX) mountant were purchased from SRL.

### Isolation of splenic phagocytes

A splenic phagocyte monolayer was prepared using the method of Steinman and Cohn (1973) and Mondal and Rai (1999). Briefly, female wall lizards were decapitated (see above) and their spleens were excised in cold 1× phosphate buffered saline (PBS, pH 7.2). The spleen of a single lizard yields 1 ml of 3×10^6^ cells ml^−1^ splenocytes. To obtain a single cell suspension of splenocytes for *in vitro* experiments conducted in the current study, the required number of spleens were pooled, macerated and passed through a nylon mesh with a pore size of 90 μm into freshly prepared cold RPMI-1640 culture media supplemented with 10% FBS. Thereafter, 500 µl of splenocyte cell suspension (3×10^6^ splenocytes ml^−1^) was loaded onto a 24-well plate and maintained in a humidified CO_2_ incubator (5% CO_2_) at 25°C. The splenic phagocytes were allowed to adhere for 90 min, after which the media containing non-adherent cells was discarded. Pilot experiments were conducted using different seeding cell counts (1.5×10^6^, 3×10^6^, 5×10^6^, 1.5×10^7^, 3×10^7^ and 5×10^7^ cells ml^−1^) to ascertain the concentration of splenocytes required to attain a final adherent phagocyte count of 1×10^6^ cells ml^−1^ in each well. Observations from the pilot experiments elucidated that when 3×10^6^ splenocytes ml^−1^ were seeded and allowed to adhere for 90 min, the final adherent splenic phagocyte population in the monolayer was 1×10^6^ cells ml^−1^ in each well.

### Assessment of cell viability

**Fig. 1.**
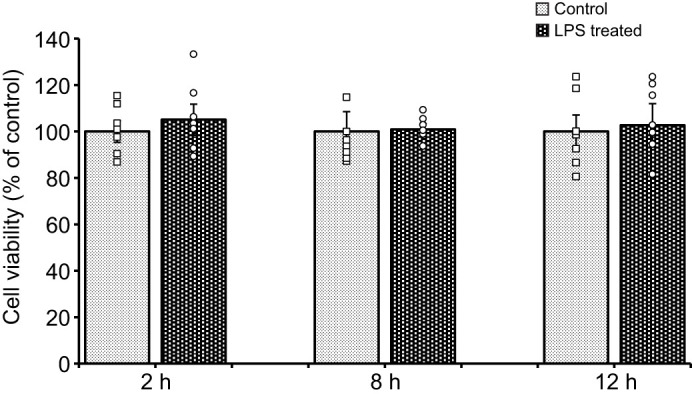
**Effect of lipopolysaccharide (LPS) treatment on the viability of wall lizard splenic phagocytes.** Viability of splenic phagocytes (1×10^6^ cells ml^−1^) exposed to 2 μg ml^−1^ LPS for different durations – 2, 8 and 12 h (*n*=8 wells/group each containing 100 μl of cell suspension) – was assessed by measuring the conversion of MTT to formazan crystals. Absorbance ratios of control and experimental groups (*A*_LPS_:*A*_control_) for each time point were plotted as percentage cell viability (absorbance ratio×100). Data for cell viability were assessed by using two-way ANOVA (*P*<0.05) followed by Tukey *post hoc* test (*P*<0.05) and are plotted as means±s.e.m.

Prior to *in vitro* experiments, the time-dependent effect of LPS on viability of splenic phagocytes was analysed using a MTT assay (Mosmann, 1983), which demonstrated that treatment with LPS for 2, 8 and 12 h had no significant effect on cell viability (Fig. 1). Briefly, 100 µl of 3×10^6^ splenocytes ml^−1^ was loaded into each well of a 96-well plate and allowed to adhere for 90 min in a humidified incubator (5% CO_2_, 25°C). Thereafter, media was discarded and wells were divided into control (media only) and experimental groups (media containing 2μg ml^−1^ LPS). The cells were incubated with or without LPS for 2, 8 and 12 h. For each group (control and experimental) of respective time points (2 h, 8 h, 12 h), 8 wells were used (*n*=8 per group). The experiment required 6 groups (control and experimental groups for each time point); hence, the total volume of cell suspension required was 100 µl×8×6=4800 μl or 4.8 ml for which approximately 5 lizards were used as the spleen of a single lizard yields 1 ml of 3×10^6^ cells ml^−1^ splenocytes required to obtain 1×10^6^ phagocytes ml^−1^. Post-incubation media was discarded, phagocytes were washed with PBS, and 50 µl of FBS-free media along with 50 µl of MTT solution (5 mg ml^−1^) was added to each well. The cells were incubated with MTT in the dark for 3 h at 25°C, after which 100 µl MTT solvent (0.1 mol l^−1^ HCl in isopropanol with 10% Triton X-100) was added to each well, incubated for 15 min and thoroughly mixed to ensure solvation of formazan crystals. Absorbance was measured at 570 nm using a BioTek Synergy H1 Multimode Reader (Agilent technologies Inc., Santa Clara, CA, USA).

### Effect of LPS-mediated immunostimulation on expression of immune-relevant genes

The splenic phagocyte monolayer was treated with 2 μg ml^−1^ LPS to assess the modulation of immune function. The culture was maintained for different durations, i.e. 2 h, 8 h and 12 h, in a humidified incubator with 5% CO_2_ at 25°C. Cells incubated with media alone for the respective time duration served as controls. All experimental and control groups consisted of 8 wells, each containing 500μl of splenocyte suspension. Thus, a total of 500 µl×8 (wells per group)×6 (number of groups)=24,000 µl or 24 ml of cell suspension was used for the *in vitro* investigation requiring the spleen from 24 lizards (1 spleen yields 1 ml of 1×10^6^ phagocytes ml^−1^). Post-incubation, the media was discarded and cells were washed with PBS. Subsequently, cells were detached from the culture plate using chilled 10 mmol l^−1^ EDTA prepared in DEPC-treated water. Cells from two wells were then pooled and centrifuged at 500 ***g*** for 10 min at 4°C to make one sample for RNA extraction; thus, for each experimental and control group, four replicates (*n*=4 per group) were used for expression analysis.

### RNA isolation and cDNA preparation

Total RNA extraction was performed using TRIzol^TM^ reagent following the manufacturer's protocol. The RNA samples were quantified using a Nanodrop spectrophotometer (ND1000, Nanodrop Technologies, Wilmington, DE, USA), and samples having an absorbance ratio (*A*_260/280_) of 1.8–2.0 were processed further for cDNA preparation. A 1 μg sample of RNA was used to prepare cDNA using the Verso cDNA synthesis kit (Thermo Fisher Scientific) following manufacturer's protocol. The cDNA prepared was verified using expression of the 18S ribosomal RNA gene (*18S rRNA*).

### Expression analysis using quantitative real time polymerase chain reaction (qPCR)

Absolute quantification of immune-relevant genes was carried out to examine the effect of LPS on lizard splenic phagocytes. Sequences of *trif* (TIR domain-containing adaptor inducing interferon-β), *myd88* (myeloid differentiation factor 88), *ap1* (activator protein 1), *nfκb* (nuclear factor kappa b), *tgfβ* (transforming growth factor β), *il1β* (interleukin 1β), *arginase* and *catalase* were obtained from the splenic transcriptome data (BioProject ID: PRJNA313009) of *H. flaviviridis* (Priyam et al., 2016). The sequences were employed to design gene-specific primers for semi-quantitative PCR (Table 1). The respective PCR products were excised, purified, commercially sequenced and verified using NCBI (National Center for Biotechnology Information) BLASTn software (https://blast.ncbi.nlm.nih.gov/Blast.cgi). The validated sequences were submitted to the NCBI database (Table 2) and used to design gene-specific primers for qPCR (Table 3). In addition to this, primers for qPCR of *tlr4* and *tlr2* (toll-like receptor 2) were taken from Priyam (2017). Each qPCR primer pair was standardised for amplification efficiency (Table 3) and single melt peak to ensure specificity. For each qPCR reaction, 6 µl of reaction mixture was prepared using 1.2 µl nuclease-free water (NFW), 3 µl SYBR^TM^ Green Universal Master mix, 0.4 µl forward and reverse primer each, as well as 1 µl cDNA. The qPCR reactions were run on a CFX Opus 384 real-time PCR system (Bio-Rad Laboratories, Hercules, CA, USA) using the thermocycling conditions as follows: hot-start at 50°C, pre-incubation at 95°C, 39 cycles of amplification and a final extension at 72°C. Each sample was run in duplicate and a no template control (NTC) was run along with the reaction to ensure the absence of non-specific amplification. For each gene, the specific PCR product was eluted, serially diluted and used as a standard during qPCR reactions. A standard curve for each gene was generated by plotting the quantification cycle (Cq) values obtained against the log concentration of each standard. This was subsequently used to extrapolate the absolute expression of the gene of interest in unknown samples.

**Table 1.** Primers for semi-quantitative PCR used to validate the sequences obtained from splenic transcriptome data of wall lizard

Gene name	Forward primer (5′ to 3′)	Reverse primer (5′ to 3′)	Annealing temperature (°C)
*ap1*	GACTCTGAATCTGGCTGA	CTCATGCGCTTCCTCTC	55.0
*nfκb*	GTGGTACGGCTCATGTTC	ACAGCCAGGAGCATCTTC	55.0
*il1β*	TGGCAGCAGATCACACTTCC	CCTGTTGAAGATGAAGCGGC	60.0
*tgfβ*	ACACAACTCACAGCACG	GCAGGATTTCACCACCA	58.1
*catalase*	ACAGTCCGTGATCCTCG	CCACAGGGATGAGAGGA	58.0
*arginase*	CTGGGCATAGCTGTGTA	ATGCCTTCACGATATGT	55.0

NCBI BioProject ID: PRJNA313009.

**Table 2.** NCBI accession numbers of gene sequences analysed in the current study

Gene	NCBI accession no.
*18S rRNA*	OP303494
*tlr2*	PV230528
*tlr4*	PV197093
*myd88*	PV231309
*trif*	PV235394
*ap1*	PV147867
*nfκb*	PV339422
*il1β*	PV156789
*tgfβ*	PV156788
*catalase*	PV137946
*arginase*	PV147866

**Table 3.** Primers for qPCR

Gene	Forward primer (5′ to 3′)	Reverse primer (5′ to 3′)	Annealing temperature (°C)	Amplification efficiency (%)	Reference
*18S rRNA*	CAAGACGGACCAGAGCG	CGGTATCTGATCGTCTTCG	59.4	100.70	Dotania et al., 2023
*tlr2*	ATCGAAAACAGCCGCAAG	GCTGGTGGGCAAAATAGAGT	57.7	100.25	Priyam, 2017
*tlr4*	CCATAGCAGCCGGAAAGTCA	AAGATGAGGCTGGCTTTGCT	60.0	107.75	Priyam, 2017
*myd88*	CGCCTTCATCTGTTACTGCC	TGACCACCACCATCCTCC	60.0	104.64	–
*trif*	AGGATGCGCTGGATAACTC	GCTGGGCATCACTCCTTTCT	62.5	94.31	–
*ap1*	ATCCTGACCTCCCCAGATGT	ACTGGGTCGGAGTAGGAGTG	60.0	94.50	–
*nfκb*	CTGGAGACAAGTGAACC	CCGCTTCCACCGTAACT	57.0	109.6	–
*il1β*	CCTACGCGACAGATTCCAT	CCCAGTGCAACAGGATTCT	60.0	96.40	–
*tgfβ*	ACACAACTCACAGCACG	GCAGGATTTCACCACCA	58.1	100.84	–
*catalase*	CACACCCAGAAGAGGAACC	TCTGGAATGCCCCGATC	56.5	91.33	–
*arginase*	TCTGGGTTGATGCACATG	GTTGTCCATGGAGGTTTC	56.5	91.83	–

### Phagocytosis assay

To study the time-dependent effect of LPS in modulating phagocytosis, 200 µl of splenic cell suspension (3×10^6^ splenocyte ml^−1^) was loaded onto the slides and allowed to adhere for 90 min at 25°C. Non-adherent cells were then washed off using PBS and slides were divided into three experimental (2 μg ml^−1^ LPS) and three control groups (media alone) for each time point (2, 8 and 12 h). Therefore, each control and LPS-treated group consisted of three slides (*n*=3 per group). The culture was maintained for varying durations in a humidified CO_2_ incubator with 5% CO_2_ at 25°C. Therefore, a total volume of 200 µl×3 (number of slides per group)×6 (number of groups)=3600 µl or 3.6 ml of cell suspension was required, obtained from the spleen of 4 lizards. Post-incubation the slides were washed with PBS (pH 7.4) and 400 µl of heat-killed yeast cell suspension (3 mg ml^−1^ PBS) was spread onto each slide. The yeast cells were allowed to be phagocytosed for 90 min after which the slides were washed thrice with PBS, fixed in methanol and stained with Giemsa. The stained cells were observed under a microscope (Nikon Eclipse E400) to assess the percentage phagocytosis and phagocytic index. The parameters of the treatment were coded to avoid any bias in counting by the experimenter. The cells were counted randomly without following any sequence or scheme; 100 phagocytes each were counted in three random fields per slide and an average of that was employed for calculations. The percentage phagocytosis and phagocytic index were calculated for each slide (Campbell et al., 2001) as follows:
(1)

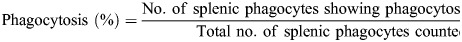

(2)

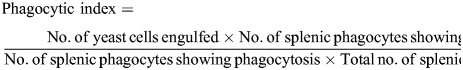


### Statistical analysis

Before employing any statistical analysis, all data were log transformed and checked for normality as well as equal variance. All statistical analyses were performed using SigmaStat software (v3.5, Systat software, Inc., San Jose, CA, USA). Data for the effect of LPS on the viability of splenic phagocytes, percentage phagocytosis and phagocytic index, as well as expression of the immune-relevant genes *tlr2*, *tlr4*, *trif*, *nfκb*, *tgfβ* and *catalase* were found to be normally distributed and were assessed using two-way analysis of variance (ANOVA; *P*<0.05) and *post hoc* pairwise comparisons were made using the Tukey *post hoc* test. While the expression of markers *myd88*, *ap1*, *il1β* and *arginase* did not meet the requirement for normality, they were still assessed by two-way ANOVA followed by Tukey *post hoc* tests as *F*-tests are robust against violations of normal distribution, especially for small sample size data, and perform equally or better than non-parametric ANOVA for non-normally distributed data (Karp et al., 2010; Schminder et al., 2010; Jones et al., 2011; Ferreira et al., 2012; Ferrari et al., 2014; Blanca et al., 2017; Floreste et al., 2022). Data are presented as means±s.e.m.

## RESULTS

### Time-dependent effect of LPS on expression of immune-relevant markers in lizard splenic phagocytes

#### TLRs and downstream effector molecules

**Fig. 2.**
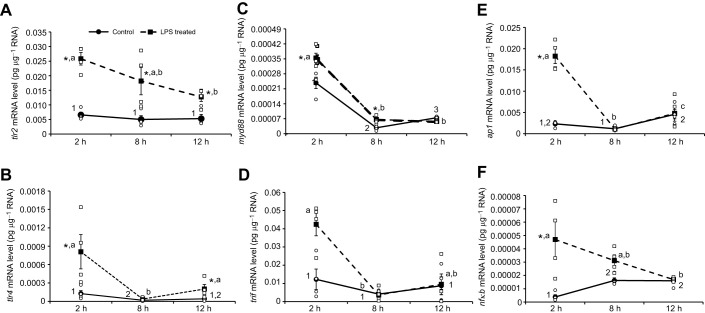
***In vitro* effect of LPS on the mRNA expression of immune-relevant markers in wall lizard splenic phagocytes.** Expression of immune-relevant receptors *tlr2* (A) and *tlr4* (B), signalling molecules *myd88* (C) and *trif* (D), and transcription factors *ap1* (E) and *nfκb* (F) following incubation with 2 μg ml^−1^ LPS for different durations (2, 8, 12 h). Filled circles and squares are means±s.e.m. for control and LPS-treated groups, respectively; open circles and squares denote individual data points for control and experimental groups, respectively (four replicates, *n*=4/group). Two-way ANOVA (*P*<0.05) was used to analyse the data, followed by Tukey *post hoc* test (*P*<0.05) for multiple comparisons. Asterisks denote significant differences in gene expression between experimental and control groups for each time point; numbers indicate significant differences within control groups; letters indicate significant differences within LPS-treated groups.

The expression of *tlr2* (*q*_2h_=5.063; 7.161; *P*<0.001; Fig. 2A) and *tlr4* (*q*_2h_=4.666; P=0.004; Fig. 2B) as well as downstream signalling molecules *myd88* (*q*_2h_=3.117; *P*=0.041; Fig. 2C), *ap1* (*q*_2h_=8.794; *P*<0.001; Fig. 2E) and *nfκb* (*q*_2h_=11.209; *P*<0.001; Fig. 2F) was found to be significantly upregulated in splenic phagocytes treated with 2μg ml^−1^ of LPS for a short duration of 2 h when compared with control. No change was observed in the expression of *trif* in LPS-treated cells in comparison to the control group (Fig. 2D). However, the expression of these effector molecules varied with a longer duration of LPS treatment, i.e. 8 h and 12 h. While *tlr2* remained significantly upregulated in LPS-treated groups even at 8 h (*q*_8h_=6.544; *P*<0.001) and 12 h (*q*_12h_=4.889; *P*=0.003) (Fig. 2A), *tlr4* demonstrated a reduction in its expression at 8 h, followed by a significant increase after 12 h (*q*_12h_=4.591; *P*=0.005) of LPS exposure (Fig. 2B). While the expression of *tlr2* showed a time-dependent reduction in LPS-treated cells from 2 h to 12 h (Fig. 2A), *tlr4* expression was significantly reduced at 8 h of LPS treatment in comparison to that at 2 h and 12 h of treatment (Fig. 2B). The downstream signalling molecule *myd88* showed a biphasic response as it was significantly (*P*<0.05) stimulated at 2 h as well as at 8 h (*q*_8h_=7.214; *P*<0.001) of LPS treatment but appeared to be inhibited at 12 h of treatment, although not significantly (Fig. 2C). However, *trif* demonstrated expression comparable to that of control after 8 and 12 h exposure to LPS (Fig. 2D). Within the LPS-treated groups, mRNA levels of both *myd88* (Fig. 2C) and *trif* (Fig. 2D) were significantly increased at 2 h and were reduced by 8 h of LPS treatment. With regard to downstream transcription factors, both *nfκb* (Fig. 2F) and *ap1* (Fig. 2E) levels in the LPS-treated cells declined to levels comparable to those of control at 8 h and 12 h. The expression of *nfκb* was also gradually reduced from 2 h to 12 h within the LPS-treated groups (Fig. 2F). A significant interaction was observed between time and treatment in the expression of *myd88* (*F*_2,18_:10.689; *P*<0.001), *ap1* (*F*_2,18_: 13.678; *P*<0.001) and *nfκb* (*F*_2,18_: 16.350; *P*<0.001). Although expression of the receptor *tlr2* and the downstream signal mediator *trif* in unstimulated splenic phagocytes remained constant at all time points, *tlr4*, *myd88*, *ap1* and *nfκb* demonstrated time-dependent differential expression even in control groups.

#### Inflammatory markers

**Fig. 3.**
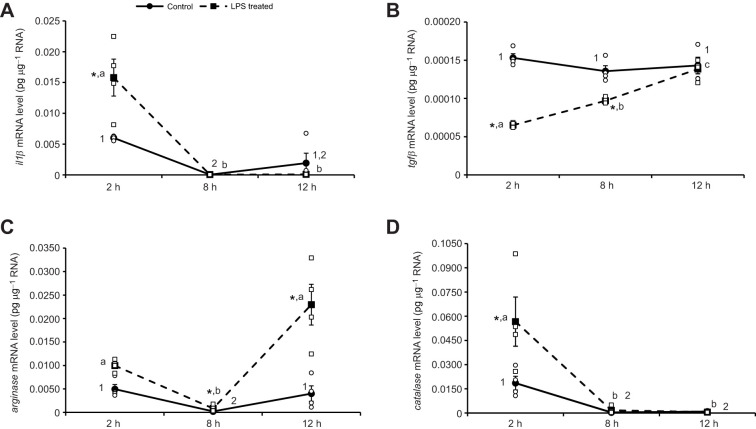
**Time-dependent modulation of inflammatory marker mRNA expression in LPS-treated wall lizard splenic phagocytes.** (A) *il1β*, (B) *tgfβ*, (c) *arginase* and (D) *catalase* expression following incubation with 2 μg ml^−1^ LPS for different durations (2, 8, 12 h). Filled circles and squares are means±s.e.m. for control and LPS-treated groups, respectively; open circles and squares denote individual data points for control and experimental groups, respectively (four replicates, *n*=4/group). Two-way ANOVA (*P*<0.05) was used to analyse the data, followed by Tukey post hoc test (*P*<0.05). Asterisks denote significant differences in gene expression between experimental and control groups for each time point; numbers indicate significant differences within control groups; letters indicate significant differences within LPS-treated groups.

Exposure to LPS for a short duration of 2 h markedly upregulated the expression of *il1β* (*q*_2h_=7.029; *P*<0.001; Fig. 3A) and *catalase* (*q*_2h_=3.231; *P*=0.035; Fig. 3D). Interestingly, while expression of *il1β* and *catalase* returned to basal levels by 8 h and 12 h of LPS exposure, expression of *arginase* was significantly higher compared with that of the respective controls at 8 h (*q*_8h_=4.283; *P*=0.007) and 12 h (*q*_12h_=7.001; *P*<0.001) of LPS treatment (Fig. 3C). However, the mRNA expression of *tgfβ* was significantly inhibited after 2 h (*q*_2h_=18.741; *P*<0.001) and 8 h (*q*_8h_=7.296; *P*<0.001) of LPS treatment (Fig. 3B). A statistically significant interaction was observed between time and treatment for expression of *il1β* (*F*_2,18_: 10.067; *P*=0.001) and *tgfβ* (*F*_2,18_: 42.000; *P*<0.001). Within the LPS-treated groups, the expression of *il1β* and *catalase* was significantly high at 2 h, then dropped to basal levels by 8 h and 12 h of treatment (Fig. 3A,D). The increased expression of *arginase* was comparable between 2 h and 12 h of LPS treatment while being lowest at 8 h of treatment (Fig. 3C). The mRNA levels of *tgfβ* demonstrated a gradual time-dependent increase in the LPS-treated groups (Fig. 3B). The expression of *il1β*, *arginase* and *catalase* demonstrated time-dependent variation in control groups: *il1β* (*q*_2h versus 8h_=4.287, *P*=0.019; *q*_2h versus 12h_=2.936, *P*=0.123; *q*_12h versus 8h_=1.351, *P*=0.614) and *arginase* (*q*_2h versus 8h_=11.239, *P*<0.001; *q*_2h versus 12h_=1.582, *P*=0.515; *q*_12h versus 8h_=9.657, *P*<0.001) expression in unstimulated splenic phagocytes was markedly low at 8 h, while that of *catalase* (*q*_2h versus 8h_=11.787, *P*<0.001; *q*_2h versus 12h_=9.183, *P*<0.001; *q*_12h versus 8h_=2.604, *P*=0.185) reached basal levels at 8 h and 12 h.

### Effect of LPS treatment on phagocytic ability of splenic phagocytes

**Fig. 4.**
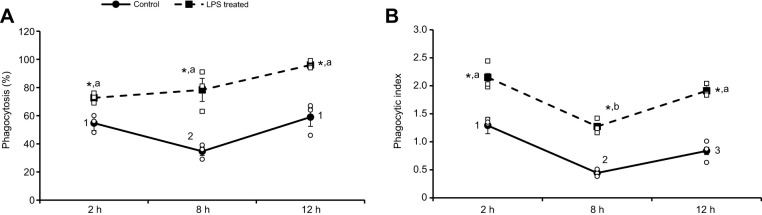
**Modulation of phagocytic activity of wall lizard splenic phagocytes in response to LPS treatment.** (A) Percentage phagocytosis and (B) phagocytic index for each time point. Filled circles and squares are means±s.e.m. for control and LPS-treated groups, respectively; open circles and squares denote individual data points for control and experimental groups, respectively (*n*=3 slides/group). Two-way ANOVA (*P*<0.05) followed by Tukey *post hoc* test (*P*<0.05) was employed to analyse the data. Asterisks denote significant differences between experimental and control groups for each time point; numbers and letters indicate significant differences in phagocytic activity at different time points for control and LPS-treated groups, respectively.

**Fig. 5.**
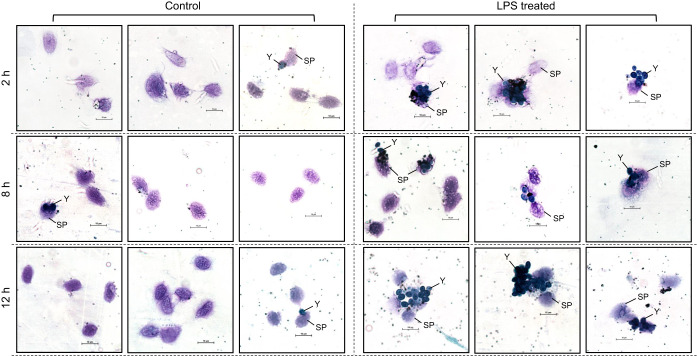
**Time-dependent effect of LPS on phagocytosis in wall lizard splenic phagocytes.** Splenic phagocytes were exposed to 2 μg ml^−1^ LPS for different durations – 2, 8 and 12 h. Cells were imaged at 1000× magnification. Representative images at each time point show yeast cells (Y) being phagocytosed by splenic phagocytes (SP). Scale bars: 10 μm.

Splenic phagocytes treated with LPS demonstrated increased phagocytosis of heat-killed yeast cells (Figs 4 and 5). The percentage phagocytosis (*q*_2h_=3.573, *P*=0.027; *q*_8h_=10.061, *P*<0.001; *q*_12h_=6.200, *P*=0.001; Fig. 4A) as well as the phagocytic index (*q*_2h_=5.896, *P*=0.001; *q*_8h_=13.680, *P*<0.001; *q*_12h_=10.894, *P*<0.001; Fig. 4B) of LPS-stimulated cells was found to be significantly higher than that of the respective control groups at all time points. A significant interaction was observed between time and treatment for percentage phagocytosis (*F*_2,12_=5.325; *P*=0.022) as well as phagocytic index (*F*_2,12_=7.778; *P*=0.007). Both parameters of phagocytosis also showed variation among control groups at different time points. While the percentage phagocytosis within the LPS-treated groups remained unchanged, the phagocytic index showed time-dependent variation. However, LPS treatment caused upregulation of phagocytosis independent of the observed trend in unstimulated splenic phagocytes.

## DISCUSSION

The current study is a pioneer attempt to elucidate the time-dependent modulation and molecular mechanism of the LPS-induced inflammatory response in splenic phagocytes of the squamate *H. flaviviridis*. Although immunostimulation by LPS is reported to induce a febrile response and heightened phagocytosis along with the modulation of various immune markers in reptiles (reviewed by Zimmerman, 2020), studies exploring the time kinetics of the reptilian immune response are lacking. A previous study from our laboratory (Mondal and Rai, 2001) demonstrated LPS-mediated stimulation of phagocytosis by wall lizard splenic phagocytes; however, the temporal dynamics of this phagocytic response remained unexplored. In the current study, phagocytic activity was observed to be significantly heightened at all time points of LPS treatment, indicating a strong pro-inflammatory response. Although percentage phagocytosis indicative of the proportion of LPS-activated phagocytes remained constant for all durations of treatment, the number of yeast cells being phagocytosed by individual phagocytes showed a biphasic pattern as evidenced by the phagocytic index of LPS-activated phagocytes, which demonstrated a peak at 2 h as well as at 12 h post-treatment while a significant decline was seen at 8 h. It is possible that after an initial burst of yeast uptake, the cells reached their maximum internalisation limit, lowering the average number of yeast particles internalised per phagocyte despite the cells still being in an active state. Bone marrow-derived macrophages of mammals are reported to display antibody-dependent cellular phagocytosis characterised by an initial engorgement phase, an attenuation phase where the cells reach maximum phagocytic capacity, followed by a hypophagia phase and a recovery phase (Pinney et al., 2020). Similarly, murine bone marrow-derived macrophages also display a biphasic trend in the engulfment of *E. coli* particles, with peak phagocytosis observed at early (4 h) and late (24 h) phagocytosis (Kapellos et al., 2016). Also, the murine macrophage cell line (RAW 264.7) is reported to demonstrate increased phagocytosis of IgG-opsonised beads for a duration of 2 h, which then dramatically reduced by 4 h (Fountain et al., 2025). Further, LPS-induced modulation of the phagocytic response at different time points has also been reported in amphibians and birds. *In vivo* LPS administration is shown to induce a time-dependent (4–24 h) progressive stimulation of phagocytic activity in turkey pulmonary macrophages and systemic phagocytes (Emery et al., 1991). Among amphibians, the percentage phagocytosis of bullfrog blood leukocytes remained unaffected after 6 h of LPS administration but significantly increased after long-term exposure of 24 h (Figueiredo et al., 2021). A similar time kinetic study in the toad *Rhinella diptycha* reported a significantly increased percentage phagocytosis after 1 h of LPS administration that declined at 3 h and became comparable to control by 6 h post-injection (Titon Junior et al., 2021). Similarly, LPS-injected *Rhinella icterica* toads displayed increased phagocytosis by blood leukocytes 2 h after injection (Titon et al., 2022). Further, peripheral monocytes from Japanese pufferfish, *Takifugu rubripes*, showed increased phagocytic activity after 5 days of *in vitro* LPS stimulation (Korenaga et al., 2013), although a time-dependent effect of LPS was not investigated.

The phagocytic response of mammalian macrophages is mediated by TLRs (Doyle et al., 2004) wherein TLR2 and TLR4 specifically recognise bacterial PAMPs such as lipoproteins and LPS, respectively (reviewed by Kawai and Akira, 2011). The splenic transcriptome analysis of *H. flaviviridis* (BioProject ID: PRJNA313009) in our laboratory has previously revealed the presence of different TLRs such as TLR2, TLR3, TLR4, TLR5, TLR7 and TLR13 (Priyam et al., 2016; Priyam, 2017). There appears to be a conservation in the TLR-mediated recognition of bacterial cell surface molecules in wall lizards as the increase in phagocytosis by LPS-stimulated splenic phagocytes coincided with the upregulation of *tlr2* and *tlr4* expression. Our results demonstrated a biphasic response of *tlr4* mRNA expression in lizard splenic phagocytes, wherein its expression significantly increased at early (2 h) and late (12 h) LPS exposure. This stimulated expression of *tlr4* could be indicative of its function as a receptor for LPS in reptiles. A study in the turtle *Pelodiscus sinensis* has previously shown that peripheral blood monocytes show an upregulation of *tlr4* mRNA upon LPS treatment, though a time-dependent progressive decline in intensity of *tlr4* stimulation was observed from 1 h to 4 h of treatment (Zhou et al., 2016). In addition, significant upregulation of splenic *tlr2* mRNA expression has been reported after 12 h of *Aeromonas hydrophila* infection in *P. sinensis*, while the expression returns to basal levels 24–72 h post-infection (Tian et al., 2024). LPS-mediated TLR4-dependent signalling has been demonstrated to require the presence of TLR2 in mammals (Good et al., 2012), suggesting that the two PRRs might be working together in the recognition of bacterial infection. Similarly, the significantly heightened expression of *tlr2* in response to LPS challenge in wall lizard splenic phagocytes in the current study indicates a possible involvement of both *tlr4* and *tlr2* in mounting an immune response.

TLR activation involves the key engagement of two adaptor proteins, namely MyD88 and TRIF for the downstream signalling mechanisms (reviewed by Takeda and Akira, 2004). TLRs employ both MyD88-dependent and MyD88-independent (TRIF-mediated) pathways (reviewed by Takeda and Akira, 2004). Upon activation by bacterial PAMPs, TLR4 employs a MyD88-dependent pathway to upregulate production of cytokines such as IL12 (interleukin 12) and TNF (tumour necrosis factor) using NFκB as a transcription factor, while TRIF is responsible for IFN-β (interferon-β) production as well as a delayed NFκB activation (reviewed by Takeda and Akira, 2004; Bagchi et al., 2007). In the current study, stimulation of *myd88* expression in lizard phagocytes at 2 h post-LPS treatment coincided with increased expression of *tlr2* and *tlr4*. In contrast, LPS treatment did not alter the levels of *trif* in the lizard splenic phagocytes. These observations could be suggestive of activation of the MyD88-dependent pathway during the early hours of bacterial exposure. Interestingly, *myd88* mRNA level remained upregulated albeit its expression remained significantly lower than that observed at 2 h, suggesting that the response to extended bacterial infection maybe modulated by a MyD88-dependent pathway. The progressive decline (from 2 h to 8 h of treatment) in *myd88* expression is suggestive of a dampening of pro-inflammatory pathways after prolonged infection. Our results gain support from a study in mandarin fish (*Siniperca chuatsi*) wherein LPS-treated head kidney lymphocytes demonstrated a significant increase in mRNA expression of *myd88* at 3, 6 and 18 h post-treatment; however, the upregulation started to decline by 18 h (Wang et al., 2021). Further, the presence of gram negative *Bacteroides* in the gut of the desert lizard *Eremias multiocellata* is known to cause enhanced expression of intestinal *myd88* along with heightened serum antibacterial activity (Yang et al., 2024). Interestingly, transcriptome profiling of salamander (*Andrias davidianus*) splenic macrophages exposed to 12 h of LPS treatment demonstrated an increased expression of *trif* while *myd88* remained unaffected (Deng et al., 2024).

TLR-mediated induction of the MyD88/TRIF pathways eventually leads to the activation of transcription factors NFκB and AP1 (reviewed by Zhang and Ghosh, 2001; reviewed by Kim et al., 2023). It is surprising that there is a complete absence of studies investigating the immune relevance of *ap1* in reptiles, while only a single study in American alligator demonstrates increased nuclear localisation of the NFκB protein upon infection with *E. coli* (Merchant et al., 2017). In line with this report, LPS-mediated enhanced expression of *nfκb* and *ap1* in lizard phagocytes was observed in the current *in vitro* study. The highest expression of *nfκb* as well as *ap1* was observed after 2 h of LPS treatment, suggesting their involvement in the early hours of infection. However, beyond 2 h, both *ap1* and *nfκb* expression returned to basal levels by 8 and 12 h of LPS exposure. The time-dependent variation in expression of these transcription factors corroborates our assumption that the pro-inflammatory pathways in lizard splenic phagocytes begin to get subdued after long-term exposure to LPS. With regard to AP1, studies in fish shed some light on the time kinetics of its expression during bacterial infection. Grass carp (*Ctenopharyngodon idella*) exposed to *A. hydrophilla* demonstrated a significant upregulation of intestinal *ap1* mRNA at 6 and 12 h of infection, which declined to basal levels at 24 h (Qu et al., 2020). Further a study in red lip mullet (*Liza haematocheila*) investigated the impact of LPS on AP1 dimer components – namely, c-Jun and c-Fos – and found their splenic mRNA expression to be biphasic as it declined 6 h post-LPS treatment, followed by a significant upregulation at 24 h, which culminated in suppression of expression after 48 h of LPS treatment (Janson et al., 2019).

LPS-treated mammalian H292 and THP1 cell lines are reported to display increased DNA binding of NFκB and AP1 as well as higher levels of pro-inflammatory cytokines (Liu et al., 2018). The upregulation of *nfκb* and *ap1* in the wall lizard splenic phagocytes following LPS challenge indicates conservation of immune pathways in reptiles, raising the possibility that these transcription factors might be involved in the upregulation of cytokine production in response to infection in lizards. Our assumption is corroborated by the increased expression of *il1β* in splenic phagocytes of wall lizards after 2 h of LPS treatment, which coincides with the heightened expression of *nfκb* and *ap1* at the same time point. The expression of *il1β* returned to basal levels by 8 and 12 h of LPS treatment, further strengthening our assumption that the pro-inflammatory pathways get subdued during later hours of infection. Similarly, LPS-treated peripheral monocytes of *P. sinensis* demonstrated an upregulation of *il1β* expression from 1 to 4 h of exposure, being maximal at 4 h (Zhou et al., 2016). In line with our study, a stimulation of *IL1β* expression was reported in the LPS-treated chicken macrophage cell line HD11 after 2 h of treatment (Keestra and van Putten, 2008). Further, a species-specific time-dependent modulation of cytokines in response to LPS was demonstrated using human and mouse macrophage cell lines, which showed heightened expression of *IL1β* mRNA following LPS challenge at all time points (2, 4, 8 and 24 h); however, maximal stimulation was observed at 2 h of LPS treatment in humans, while in mouse macrophages it occurred at 4 h (Huang et al., 2012). Similar temporal variation in *il1β* expression during infection has also been reported in other non-mammalian vertebrates such as amphibians (Floreste et al., 2022), fishes (Wiens and Vallejo, 2010; Holen et al., 2012; Korenaga et al., 2013) and birds (Keestra and van Putten, 2008). LPS-injected *R. diptycha* demonstrated increased splenic mRNA expression of *il1β* at 1 and 18 h while no change was observed at 3 and 6 h (Floreste et al., 2022). Similarly, LPS-treated Japanese pufferfish monocytes showed a time-dependent increase in *il1β* mRNA expression from 1 to 12 h, returning to basal levels by 24 h (Korenaga et al., 2013). Further, LPS-treated cod head kidney cells (Holen et al., 2012) as well as spleens from rainbow trout (Wiens and Vallejo, 2010) infected with *Yersinia ruckeri* demonstrated increased *il1β* expression within 1 day of immune challenge. The increase in splenic *il1β* expression in *Y. ruckeri*-infected rainbow trout reached maximal levels at day 3, and remained high at days 5 and 7 (Wiens and Vallejo, 2010). It is noteworthy that the early spike in *il1β* mRNA expression coincided with a significant increase in phagocytic activity of wall lizard splenic phagocytes. Studies in rat peritoneal macrophages (Bergman et al., 2002) as well as murine microglial cell lines (Ferreira et al., 2011) have established a positive interlink between IL1β production and phagocytosis in the presence of LPS. Our results suggest a similar interplay during the early hours of LPS challenge in reptiles.

In addition to a pro-inflammatory pathway, we also investigated the effect of LPS on the expression of the anti-inflammatory cytokine *tgfβ*. LPS exposure was seen to induce a time-dependent reduction in mRNA expression of the cytokine in wall lizard splenic phagocytes at 2 h and 8 h, which eventually returned to basal levels by 12 h, suggesting a strong suppression of the anti-inflammatory response during the early hours of infection. Furthermore, at 12 h of LPS treatment, the return of *tgfβ* to basal levels coincided with a reduction of *myd88* to basal levels, which possibly hints at a TGFβ-mediated inhibition of the pro-inflammatory response in lizard phagocytes. This is supported by reports in mammals wherein TGFβ is shown to induce inhibition of MyD88 and its downstream signalling pathway (reviewed by Naiki et al., 2005). In addition, a probable interplay exists between the two cytokines *il1β* and *tgfβ* in squamates, as our results demonstrated an early increase in *il1β* mRNA expression which coincided with a significant reduction of the anti-inflammatory cytokine *tgfβ*. The cytokines IL1β and TGFβ often function antagonistically in mammals (reviewed by Wilson, 2021). This trend is also conserved in grass carp (*C. idella*), wherein TGFβ caused a direct inhibition of LPS-induced *il1β* production by head kidney leukocytes (Wang et al., 2016). A separate study in grass carp demonstrated an inverse temporal relationship between the extent of upregulation of *il1β* and *tgfβ* in response to LPS treatment, wherein *il1β* was maximally stimulated during early exposure (6 h) while *tgfβ* was majorly upregulated only after extended LPS exposure (12 h), at which point the extent of *il1β* stimulation was reduced (Wei et al., 2015). The time-dependent pattern of *tgfβ* expression in LPS-stimulated lizard splenic phagocytes observed in the current study gains support from a study in rainbow trout wherein LPS stimulation of head kidney macrophages led to the downregulation of *tgf-β1a* expression, while its paralogue *tgf-β1b* was upregulated at all time points (4, 8 and 24 h) of treatment (Maehr et al., 2013). However, contrary reports are also available wherein *tgfβ* expression is upregulated after 6 and 12 h of LPS infection in grass carp macrophages (Wei et al., 2015) while in goldfish macrophages it is stimulated at 24 h of LPS treatment (Haddad et al., 2008). Contrary to our results, chicken heterophils exposed to the bacteria *Salmonella enteritidis* also demonstrated an increased expression of *TGFβ4* (Kogut et al., 2003)*.*

LPS-mediated stimulation of macrophages causes increased production of reactive oxygen species which counteract oxidative stress (reviewed by Tan et al., 2016). Catalase is an enzyme involved in the conversion of hydrogen peroxide to water and oxygen molecules, thereby preventing oxidative alteration of cellular nucleic acids, proteins and lipids (reviewed by Nandi et al., 2019). While catalase activity has been reported to increase in spleen after intraperitoneal hydrogen peroxide treatment in turtles (Zhang et al., 2019), its expression post-bacterial infection in reptiles remains elusive. In the present study, the mRNA expression of this enzyme in wall lizard splenic phagocytes was significantly increased at 2 h of LPS treatment, suggestive of an early anti-oxidant response, which drops to basal levels after 8 h and 12 h of infection. In line with our study, the mRNA expression of *catalase* in yellowtail clownfish (*Amphiprion clarkii*) showed temporal variation in the presence of LPS as it significantly increased in the peripheral blood cells at 6 and 12 h of LPS treatment, followed by a return to basal levels at 24 h and 48 h, with a subsequent upregulation at 72 h post-LPS challenge (Sandamalika et al., 2021). Also, a study in LPS-infected rock bream (*Oplegnathus fasciatus*) extending from 0 to 48 h demonstrated a significant increase in *catalase* expression in blood cells only at 12 h (Elvitigala et al., 2013). However, LPS induced a decline in catalase activity in lymphocytes of the northern snakehead (*Channa argus*) (Li et al., 2020) as well as the serum of broiler chickens (Gao et al., 2018) after 24 h of exposure.

As LPS is known to induce the expression of *arginase* in mammalian macrophages (Corraliza et al., 1995; Wang et al., 1995; Sonoki et al., 1997; Sosroseno et al., 2006), the current study also investigated the effect of LPS on the expression of *arginase* in lizard splenic phagocytes. Arginase is a key enzyme involved in the conversion of l-arginine to ornithine and urea that aid the production of polyamines and proline involved in cell repair and proliferation (reviewed by Munder, 2009). In mammals, it is expressed in response to different PAMPs including LPS and serves as an endogenous immunosuppressive agent. Although studies investigating the role of arginase in reptilian immunity are lacking, a single study on the regenerating tail of the lizard *Podarcis muralis* revealed the presence of arginase-expressing macrophages in blastema that the authors predicted had role in wound healing (Alibardi, 2020). Nonetheless, arginase has been reported in the liver as well as kidney of several species of turtles, snakes and lizards including *H. flaviviridis* (reviewed by Khalil and Haggag, 1960; Baby et al., 1976). Interestingly, in the present study, *arginase* expression in lizard splenic phagocytes remained significantly higher than that of the control at 8 h and 12 h of LPS treatment. The maximal expression of *arginase* at 12 h could be indicative of the activation of cell repair in the lizard phagocytes. Similar results have been reported in rat peritoneal macrophages, wherein LPS treatment could induce detectable mRNA levels of *arginase* after 4 h of LPS treatment that peaked by 12 h (Sonoki et al., 1997). Also, while the head kidney cells from Atlantic salmon showed a marked increase in the expression of *arginase* upon treatment with LPS for 24 h (Martins et al., 2019), head kidney leukocytes from largemouth bass displayed stimulation of *arginase* at 6 h post-LPS challenge with no observable change at 3 and 24 h of treatment (Qian et al., 2024).

**Fig. 6.**
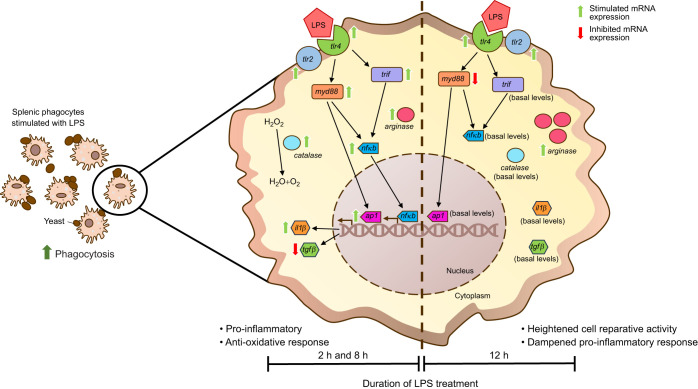
**Temporal dynamics and molecular mechanisms of LPS-induced modulation of the innate immune response in wall lizard splenic phagocytes.** *In vitro* treatment of splenic phagocytes with LPS led to an enhanced phagocytic activity as well as upregulation of *tlr4* expression, which may serve as the primary receptor for LPS. In addition, LPS-induced stimulation of *tlr2* expression is indicative of its role in assisting TLR4-mediated LPS recognition. During the early hours (2 and 8 h) of LPS treatment, the phagocytes displayed upregulation of signalling molecules *myd88* and *trif* as well as transcription factors *nfκb* and *ap1*, which returned to basal levels by 12 h. The activation of both MyD88-dependent and TRIF-mediated pathways would trigger the activation of a pro-inflammatory response as evidenced by increased *il1β* mRNA expression. Additionally, the early hours of LPS treatment were characterised by increased expression of the anti-oxidative enzyme *catalase* and inhibition of expression of the anti-inflammatory cytokine *tgfβ*. By 12 h of LPS exposure, the response shifted towards cell repair marked by increased expression of *arginase*.

The findings of our current study indicate conservation of LPS-mediated activation of the TLR pathway in wall lizard splenic phagocytes. LPS challenge in these reptilian phagocytes drives a time-dependent increase in phagocytosis with altered expression of different immune-relevant genes (Fig. 6). The increased phagocytosis by these immune cells during early and late LPS challenge can be attributed to the biphasic upregulation of *tlr4* at 2 h and 12 h, which could be a possible receptor for LPS in wall lizards. Furthermore, the heightened expression of *tlr2* at all time points suggests that both TLR4 and TLR2 might be important for LPS-mediated activation of the inflammatory response. Also, the results of our study indicate that lizard TLR4/2 might be employing both MyD88 and TRIF as downstream effector molecules to upregulate the expression of the transcription factors NFKβ and AP1, which would eventually upregulate the pro-inflammatory response by stimulating the expression of cytokines such as IL1β. Furthermore, there appears to be a conservation in the suppression of the anti-inflammatory cytokine TGFβ during the early hours of LPS challenge, while the later hours of LPS treatment are characterised by the cell reparative action of arginase. In addition, the oxidative stress caused by LPS in splenic phagocytes appears to be counteracted by catalase. The current study provides valuable insights into the molecular mechanism of the immune response in splenic phagocytes of wall lizards, thus allowing for advancement in our understanding of the reptilian immune system.
